# Value-Based Pricing of Resmetirom for Metabolic Dysfunction–Associated Steatotic Liver Disease

**DOI:** 10.1001/jamanetworkopen.2025.17122

**Published:** 2025-06-27

**Authors:** Phuc Le, Srinivasan Dasarathy, William H. Herman, Olajide A. Adekunle, Ha T. Tran, Victoria Criswell, Wen Ye, Nicole Welch, Yihua Yue, Michael B. Rothberg

**Affiliations:** 1 Center for Value-Based Care Research, Primary Care Institute, Cleveland Clinic, Cleveland, Ohio; 2 Department of Gastroenterology, Hepatology, and Nutrition, Digestive Disease and Surgery Institute, Cleveland Clinic, Cleveland, Ohio; 3 Department of Inflammation and Immunity, Lerner Research Institute, Cleveland, Ohio; 4 University of Michigan School of Public Health, Ann Arbor; 5 University of Michigan School of Medicine, Ann Arbor

## Abstract

**Importance:**

Resmetirom can slow fibrosis progression and improve resolution of metabolic dysfunction–associated steatohepatitis (MASH). As the first Food and Drug Administration–approved pharmacologic treatment for MASH and fibrosis stages F2 and F3, resmetirom is very expensive, potentially creating a financial barrier to patient access.

**Objective:**

To estimate the cost-effectiveness of resmetirom vs standard of care (SoC) over a lifetime and to determine a price threshold at which resmetirom would be cost-effective.

**Design, Setting, and Participants:**

This economic evaluation used an agent-based state-transition microsimulation model with a yearly cycle and 14 distinct histologically defined health states: metabolic dysfunction–associated steatotic liver disease (MALSD) or F0, MASH, fibrosis stages F1 to F3 with or without MASH, cirrhosis, decompensated cirrhosis, liver cancer, liver transplant, and liver-related and other-cause death. The simulated cohort included US adults who entered the model with MASH and fibrosis stage F2 or F3. The study was conducted from March to December 2024.

**Exposures:**

Resmetirom vs SoC.

**Main Outcomes and Measures:**

Costs (in 2023 US dollars), quality-adjusted life-years (QALYs), and the incremental cost-effectiveness ratio (ICER) (calculated as incremental costs divided by incremental QALYs).

**Results:**

A cohort of 200 000 patients (mean [SD] age, 57.1 [10.5] years; 89 000 [44.5%] male; 132 600 [66.2%] with MASH-F3) was simulated. Compared with SoC, resmetirom was associated with a gain of 0.26 QALYs/patient. Lifetime drug cost was $54 754, and treatment saved $18 499 in MASLD-related medical costs for a total incremental cost of $36 255 per patient. The ICER was $140 134/QALY. At willingness-to-pay (WTP) thresholds of $50 000/QALY, $100 000/QALY, and $150 000/QALY, the maximal annual prices of resmetirom were $10 914, $15 406, and $19 879, respectively. The lower the discontinuation rate, the greater the cost-effectiveness and medical cost savings, but the higher the total treatment costs. Without discontinuation, the ICER would be $318 740/QALY, supporting a price between $5645 and $10 619 per year at WTP thresholds of $50 000/QALY and $150 000/QALY, respectively.

**Conclusions and Relevance:**

In this economic evaluation, resmetirom was not cost-effective at a $100 000/QALY WTP threshold. The conclusion was sensitive to model assumptions, especially the discontinuation rate. More data on the long-term effectiveness of resmetirom on MASLD progression and non-MASLD complications would be necessary to accurately determine the economic value of resmetirom.

## Key Points

**Question:**

Compared with standard of care, is resmetirom cost-effective in treating metabolic dysfunction–associated steatohepatitis and significant fibrosis among US adults in a simulated cohort?

**Findings:**

In this economic evaluation, at a price of $19 011/year, resmetirom was not cost-effective compared with standard-of-care treatment at a $100 000/quality-adjusted life-year willingness-to-pay threshold. This conclusion was sensitive to the discontinuation rate and other model assumptions.

**Meanings:**

These findings suggest that resmetirom does not provide good value for money at its current price; however, given the sensitivity of cost-effectiveness to the discontinuation rate and other variables, better data about patient adherence and clinical treatment efficacy are important.

## Teaser

This economic evaluation examines the cost-effectiveness of resmetirom for the treatment of metabolic dysfunction–associated steatotic liver disease (MASLD) and metabolic dysfunction–associated steatohepatitis, calculating the price threshold at which this drug would be cost-effective.

## Introduction

Metabolic dysfunction–associated steatotic liver disease (MASLD), formerly known as nonalcoholic fatty liver disease (NAFLD), affects one-third of global adults.^1^ One-fifth of patients have metabolic dysfunction–associated steatohepatitis (MASH), formerly known as nonalcoholic steatohepatitis (NASH), which may lead to cirrhosis, hepatocellular carcinoma (HCC), liver transplant (LT), and death.^2^ The economic burden of MASLD is substantial, driven by lost productivity and diminished quality of life.^3,4,5^ MASH is now the second leading cause of LT in the US.^6,7^

In 2024, a phase 3 randomized clinical trial (RCT)^8^ demonstrated that resmetirom slowed fibrosis progression, improved MASH resolution, and decreased low-density lipoprotein cholesterol (LDL) levels compared with placebo. Therefore, the Food and Drug Administration approved resmetirom in March 2024, making it the first approved medication for MASH and fibrosis stages F2 and F3.^9^ As such, it is very expensive. Although prices paid by insurers are proprietary, the average annual wholesale price on the UpToDate Lexidrug database is $57 670,^10^ creating a potential barrier to patient access.

As multiple stakeholders navigate MASLD management in a cost-constrained environment, understanding the economic value of resmetirom is important. Previous analyses,^11,12,13^ assuming a drug price of $19 011/year, have produced incremental cost-effectiveness ratios (ICERs) ranging from cost-saving to $53 000/quality-adjusted life-year (QALY) and have suggested an annual drug price as high as $55 000 could be cost-effective. Such estimates depend on assumptions about treatment effectiveness and MASLD’s natural history, which are evolving. To inform pricing strategies and health care policy decisions, we estimated the cost-effectiveness of resmetirom using recent data on MASLD’s natural history and treatment efficacy. We also determined the price threshold at which resmetirom would be considered cost-effective in the United States.

## Methods

### Model Overview

**Figure 1.**
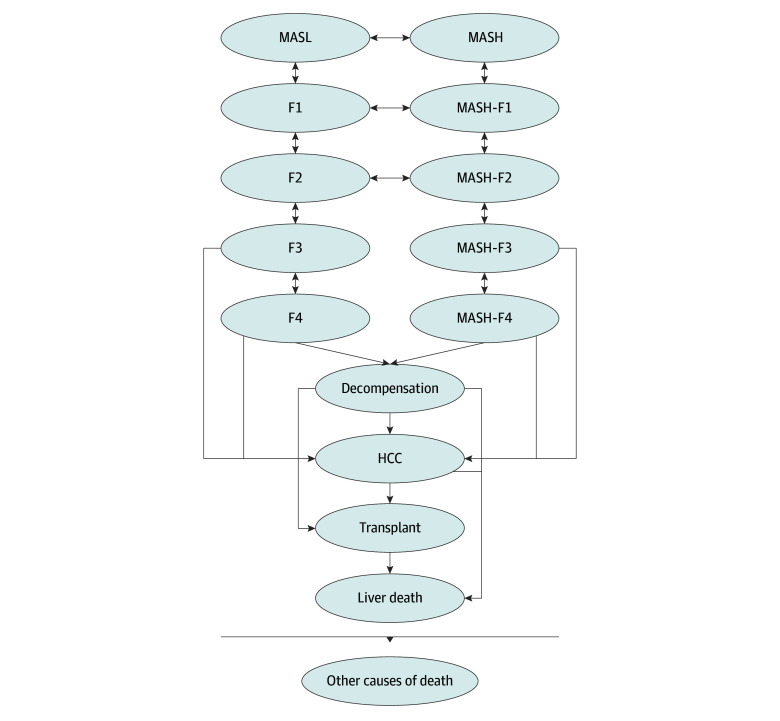
Model Diagram of the Progression of Metabolic Dysfunction–Associated Steatotic Liver Disease In each cycle, people in any health state can die from nonliver causes. F0 to F4 indicates fibrosis stages 0 (no fibrosis) to 4 (cirrhosis); HCC, hepatocellular carcinoma; MASL, metabolic dysfunction–associated steatotic liver; and MASH, metabolic dysfunction–associated steatohepatitis.

We developed an agent-based state transition model with a yearly cycle to compare the cost-effectiveness of resmetirom vs standard of care (SoC) in adults with MASH and fibrosis stages F2 or F3 (Figure 1).^14^ The model included 14 mutually exclusive health states: metabolic dysfunction–associated steatotic liver (MASL) or F0; MASH; F1 to F3, with or without MASH; compensated cirrhosis (F4), with or without MASH; decompensated cirrhosis (DC); HCC; LT; liver-related death; and death from other causes. We simulated 200 000 patients receiving nutrition and exercise counseling with age, sex, and fibrosis stage distribution reflecting participants in the MAESTRO-NASH trial.^8^ Patients entered the model in MASH-F2 or MASH-F3 state, then transitioned between states annually until all died. Compared with SoC, the resmetirom group had increased probability of fibrosis regression and MASH resolution and decreased risk of fibrosis progression. Because resmetirom was only approved for MASH-F2 and MASH-F3, treatment was discontinued once patients developed cirrhosis or worse. Although resmetirom decreases LDL levels, its impact on cardiovascular disease (CVD) is unknown. Therefore, we chose not to include CVD events in the model. Model outputs included costs and estimated effectiveness (cases of DC, HCC, LT, liver-related and all-cause deaths and QALYs). The ICER between strategies was calculated as incremental costs divided by incremental QALYs. The analysis was conducted from the payer perspective, with costs and QALYs discounted at 3%/year. Costs were presented in 2023 US dollars, adjusted for inflation using the US Consumer Price Index–Medical Care Component. The model was developed in AnyLogic University version 8 (AnyLogic Inc). We reported the study following the Consolidated Health Economic Evaluation Reporting Standards (CHEERS) guideline.^15^ Because this study used published and deidentified data, it did not constitute human participant research, per the Common Rule.

### Model Inputs, Data Sources, and Assumptions

We reviewed US-based epidemiological studies to identify model inputs. Point estimates and ranges for sensitivity analysis appear in Table 1.^5,8,16,17,18,19,20,21,22,23,24,25,26,27,28,29,30^

**Table 1.** Model Inputs

Inputs	Base-case value	Range for 1-way sensitivity analysis	Reference
Patient characteristics			
Sample size for simulation, No.	200 000	NA	Harrison et al,^8^ 2024
Age, mean (SD), y	57.1 (10.5)	NA	Harrison et al,^8^ 2024
Sex, No. (%)			
Female	111 000 (55.5)	NA	Harrison et al,^8^ 2024
Male	89 000 (44.5)		
Initial fibrosis stage distribution, No. (%)			
MASH-F2	67 400 (33.7)	NA	Harrison et al,^8^ 2024
MASH-F3	132 600 (66.2)		
Transition probabilities			
No MASH to MASH	0.006	0.0045-0.0075	Le et al,^16^ 2022
MASH to no MASH	0.013	0.0098-0.0163	Le et al,^16^ 2022
Among MASL			
F0 to F1	0.0662	0.0556-0.0766	Le et al,^16^ 2022
F1 to F0	0.0458	0.0371-0.0543	Le et al,^16^ 2022
F1 to F2	0.0741	0.0634-0.0848	Le et al,^16^ 2022
F2 to F1	0.0778	0.0638-0.0916	Le et al,^16^ 2022
F2 to F3	0.0690	0.0557-0.0821	Le et al,^16^ 2022
F3 to F2	0.0948	0.0806-0.1088	Le et al,^16^ 2022
F3 to F4	0.0605	0.0489-0.0720	Le et al,^16^ 2022
F4 to F3	0.0768	0.0557-0.0974	Le et al,^16^ 2022
Among patients with MASH			
MASH to MASH-F1	0.0984	0.0644-0.1312	Le et al,^16^ 2022
MASH-F1 to MASH	0.0428	0.0284-0.0569	Le et al,^16^ 2022
MASH-F1 to MASH-F2	0.0981	0.0772-0.1186	Le et al,^16^ 2022
MASH-F2 to MASH-F1	0.0638	0.0452-0.0821	Le et al,^16^ 2022
MASH-F2 to MASH-F3	0.0907	0.0689-0.1120	Le et al,^16^ 2022
MASH-F3 to MASH-F2	0.0948	0.0806-0.1088	Le et al,^16^ 2022
MASH-F3 to MASH-F4	0.0779	0.0599-0.0957	Le et al,^16^ 2022
MASH-F4 to MASH-F3	0.0766	0.0519-0.1007	Le et al,^16^ 2022
F4 or MASH-F4 to DC	0.0411	0.0410-0.0795	Sanyal et al,^17^ 2006 and Chhatwal et al,^18^ 2022
F3 or MASH-F3 to HCC	0.0011	0.0004-0.0018	Sanyal et al,^19^ 2021
F4 or MASH-F4 to HCC	0.0069	0.0022-0.037	Rustgi et al,^20^ 2022
DC to HCC	0.0069	0.0022-0.037	Assumption
DC to LT	0.0300	0.0230-0.040	Assumption
HCC to LT	0.0300	0.0230-0.040	Rustgi et al,^20^ 2022
Mortality			
Background	Age- and sex-specific	NA	CDC^21^
Increased risk of death in MASLD	1.15	NA	Sanyal et al,^19^ 2021
DC	0.0734	0.0689-0.0779	Nyberg et al,^22^ 2020; Vilar-Gomez et al,^23^ 2018; and Sanyal et al,^17^ 2006
HCC	Varied by years since diagnosis	NA	SEER^24^
LT	Varied by age at transplant and years since transplant	NA	OPTN^25^
Costs, 2023 $			
Annual drug cost	19 011.00	NA	Javanbakht et al,^26^ 2023 and Tice et al,^27^ 2023
Annual health state cost			
F0	0	NA	Assumption
F1	248.94	186.71-311.18	Younossi et al,^5^ 2019 and assumption
F2	248.94	186.71-311.18	Younossi et al,^5^ 2019 and assumption
F3	613.00	460.05-766.75	Younossi et al,^5^ 2019 and assumption
MASH	498.00	373.41-622.36	Younossi et al,^5^ 2019
MASH-F1	498.00	373.41-622.36	Younossi et al,^5^ 2019
MASH-F2	498.00	373.41-622.36	Younossi et al,^5^ 2019
MASH-F3	613.00	460.05-766.75	Younossi et al,^5^ 2019
F4 or MASH-F4	21 836.00	16 377.34-27 295.56	Younossi et al,^5^ 2019 and Kaplan et al,^28^ 2018
DC	41 203.00	30 902.33-51 503.89	Younossi et al,^5^ 2019 and Kaplan et al,^28^ 2018
HCC	107 696.00	80 771.64-134 619.40	Younossi et al,^5^ 2019 and Kaplan et al,^28^ 2018
LT procedure and year 1 after LT	466 505.00	349 878.74-583 131.24	Younossi et al,^5^ 2019 McAdam-Marx et al,^29^ 2011
Post-LT (annual cost after year 1)	14 828.00	11 120.96-18 534.93	Younossi et al,^5^ 2019
Utility			
F0	1.00	0.85-1.00	Rustgi et al,^20^ 2022
F1	1.00	0.84-1.00	Rustgi et al,^20^ 2022
F2	1.00	0.84-1.00	Rustgi et al,^20^ 2022
F3	0.84	0.76-0.92	Rustgi et al,^20^ 2022
MASH	0.85	0.77-0.94	Rustgi et al,^20^ 2022
MASH-F1	0.84	0.76-0.92	Rustgi et al,^20^ 2022
MASH-F2	0.84	0.76-0.92	Rustgi et al,^20^ 2022
MASH-F3	0.84	0.76-0.92	Rustgi et al,^20^ 2022
F4 or MASH-F4	0.80	0.72-0.88	Rustgi et al,^20^ 2022
DC	0.60	0.54-0.66	Rustgi et al,^20^ 2022
HCC	0.72	0.65-0.79	Rustgi et al,^20^ 2022
LT	0.73	0.66-0.80	Rustgi et al,^20^ 2022
Post-LT	0.80	0.72-0.88	Rustgi et al,^20^ 2022
Treatment-related parameter			
Treatment effectiveness			
Fibrosis improvement, RR			
MASH-F2	2.13	1.36-2.89	Harrison et al,^8^ 2024
MASH-F3	1.54	1.02-2.31	Harrison et al,^8^ 2024
Fibrosis worsening, RR			
MASH-F2	0.54	0.38-0.78	Harrison et al,^8^ 2024
MASH-F3	0.69	0.49-0.96	Harrison et al,^8^ 2024
MASH resolution, RR	2.87	2.01-4.10	Harrison et al,^8^ 2024
All-cause discontinuation, %	0.213	0.000-0.500	Harrison et al,^30^ 2023

Abbreviations: CDC, US Centers for Disease Control and Prevention; DC, decompensated cirrhosis; F0, fibrosis stage 0; F1, fibrosis stage 1; F2, fibrosis stage 2; F3, fibrosis stage 3; F4, fibrosis stage 4 or cirrhosis; HCC, hepatocellular carcinoma; LT, liver transplant; MASH, metabolic dysfunction-associated steatohepatitis; MASL, metabolic dysfunction-associated steatotic liver; NA, not applicable; OPTN, Organ Procurement and Transplantation Network; RR, relative risk; SEER, Surveillance, Epidemiology, and End Results.

#### Natural History of MASLD

Rates of fibrosis progression and regression as well as of MASH resolution and development were derived from our meta-analysis of paired liver biopsies.^16^ Rates from observational studies supplied base-case estimates, and those from RCTs were used for scenario analysis. Because no studies stratified rates of MASH resolution and development by fibrosis stage, we assumed uniform rates for stages F0 to F3. The model did not distinguish MASH vs non-MASH once patients developed cirrhosis because it is hard to distinguish them via liver biopsies.

Development of HCC from F3 was based on pooled longitudinal studies^19,22,23,31^; development of HCC from F4 was based on a previous cost-effectiveness analysis.^20^ Once patients developed DC, we assumed that they could not regress to cirrhosis, and the transition from cirrhosis to DC was independent of MASH status.^11,12,19,22,31^ As patients with DC had a low rate of HCC,^19,22^ we assumed they transitioned to HCC at a rate similar to that from F4. The rate of LT from HCC was similar to that from DC, which were estimated following Rustgi et al.^20^

Liver-related mortality could occur from HCC, DC, and LT. We derived HCC mortality from the Surveillance, Epidemiology, and End Results program^24^ and DC mortality from longitudinal studies.^17,22,23^ We estimated LT mortality by age at transplant and year after transplant using the United Network for Organ Sharing data.^25^ Age- and sex-specific background mortality was based on US 2019 life table and adjusted for increased mortality risk in MASLD.^19,21^ The model did not track deaths from CVD or other competing risks because they were already accounted for in background mortality.

#### Treatment-Related Parameters

Patients in the SoC strategy had transition probabilities as described in the previous natural history section. Patients taking resmetirom had slower fibrosis progression rates and faster regression rates as well as faster rates of MASH resolution compared to SoC patients. Based on the RCT,^8^ efficacy was expressed as relative risks (RRs) of fibrosis improvement and worsening for patients with F2 and F3 separately. To estimate the RR of fibrosis worsening among patients with MASH-F3, we used the percentage of patients with a 25% or greater increase in liver stiffness between baseline and week 52.^8^ We assumed patients with fibrosis stages F0 to F2 who continued treatment had the same RRs of fibrosis improvement and worsening and applied the same RR of MASH resolution for all fibrosis stages. Treatment was assumed to continue for life unless discontinued due to adverse events or other reasons or when patients entered stage F4. If patients improved from stage F4 to F3, they would restart treatment and continue to accrue benefits as before. We aggregated data for both resmetirom dosages when estimating efficacy.

In the base case, patients discontinued treatment at a constant rate of 21.3%/year.^30^ We conducted scenario analysis with discontinuation due to adverse events only (4.3%/year).^8^ We did not model adverse events because there was no difference in serious adverse events between resmetirom vs placebo.

#### Costs

Costs of MASLD-related health states were based on the literature and adjusted for inflation.^5,28,29^ We assumed that stage F3 cost the same as MASH-F3, F1 or F2 cost half as much as MASH-F1 or MASH-F2, and MASL had no cost. For the base-case, drug cost was based on the manufacturer’s suggested price ($19 011/year).^26,27^ We did not use the wholesale drug price because most insurance plans would negotiate a discount.^32^

### Statistical Analysis

#### QALYs

We estimated QALYs from published utility weights. We derived MASLD-related utilities from a previous economic evaluation.^20^ The 36-Item Short Form Survey scores collected from patients with NAFLD and NASH were transformed into the health utility index and applied to MASH with fibrosis stages F0 to F3 health states.^33,34^ Because there were no data available for non-MASH fibrosis health states, we assumed that people with fibrosis stage F0 to F2 without MASH did not have a reduction in quality of life, while those with stage F3 had the same utility as those with MASH-F3. For cirrhosis, DC, HCC, and LT, we drew utilities from patients with hepatitis C.^35^ The utility after LT was derived from the 15-dimensional instrument answered by first-time transplant patients.^36^ All MASLD-related utilities were adjusted for age- and sex-specific baseline utilities.^37^ Finally, we did not assign a disutility for taking resmetirom in the base case, but varied it from 0.001 to 0.004 QALYs/year in sensitivity analysis.

#### Sensitivity Analysis

We conducted threshold analysis to determine the maximum price at which resmetirom was cost-effective at $50 000/QALY gained, $100 000/QALY gained, and $150 000/QALY gained. We also varied the discontinuation rate from 0% to 50% and other model inputs within their 95% CIs or possible ranges. Other analyses included applying transition probabilities for disease progression estimated from RCTs^16^ and adding disutility for taking resmetirom. Since resmetirom could provide different benefits to patients with coexisting MASLD and type 2 diabetes (T2D), whose risks of advanced liver outcomes are higher than those with MASLD alone, we conducted a scenario analysis in which we assumed disease progressed faster in patients with MASLD and T2D (eAppendix, eTable 1, and eTable 2 in Supplement 1).

#### Model Validation

We validated model outputs against data from the NASH Clinical Research Network (CRN).^19^ To do this, we created a hypothetical patient cohort of 200 000 patients (mean age, 52 years; 35.6% male) with a distribution of fibrosis stages and MASH status similar to the patients in NASH CRN. We generated 8-year follow-up data for this hypothetical cohort and compared these model-estimated outcomes vs observed outcomes from NASH CRN.

## Results

### Model Validation

For a cohort of patients similar to those in NASH CRN, our model produced an 8-year survival curve that closely matched observed survival (eFigure 1 in Supplement 1). The modeled HCC and liver-related death incidence (9.5 and 16.7 per 10 000 person-years, respectively) closely approximated that among NASH CRN participants (11.0 [95% CI, 3.8-18.3] per 10 000 person-years and 14.8 [95% CI, 6.4-23.1] per 10 000 person-years, respectively).

### Base-Case Results and Price Threshold Analysis

The model simulated 200 000 patients (mean [SD] age, 57.1 [10.5] years; 89 000 [44.5%] male; 132 600 [66.2%] with MASH-F3). For every 1000 patients, resmetirom treatment was estimated to prevent 8 HCC cases, 6 LTs, and 24 liver-related deaths over a lifetime. Compared with SoC, resmetirom led to a gain of 0.26 discounted QALYs/patient (Table 2). The drug cost $54 754, and treatment saved $18 499 in MASLD-related medical costs, resulting in a total discounted lifetime incremental cost of $36 255/patient. The ICER of resmetirom vs SoC was $140 134/QALY. To be cost-effective at $50 000, $100 000 and $150 000/QALY, the annual price could not exceed $10 914, $15 406, and $19 879, respectively (Table 3).

**Table 2.** Discounted Lifetime Costs and Estimated Effectiveness of Treatment With Resmetirom in Patients With MASH-F2 or MASH-F3^a^

Patient group	Medical costs, $^b^	Drug costs, $^c^	Total costs, $	Incremental total costs, $	QALYs	Incremental QALYs	ICER, $/QALY
SoC	92 908	0	92 908	NA	10.48	NA	NA
Resmetirom	74 408	54 754	129 163	36 255	10.74	0.26	140 134

Abbreviations: F2, fibrosis stage 2; F3, fibrosis stage 3; ICER, incremental cost-effectiveness ratio; MASH, metabolic dysfunction–associated steatohepatitis; QALY, quality-adjusted life-year; SoC, standard of care.

^a^ Costs and effectiveness were both discounted at 3%/year.

^b^ Medical costs included all costs associated with treating metabolic dysfunction–associated steatotic liver disease–related health states.

^c^ Drug costs included cost of resmetirom added up for the years during which patients were treated.

**Table 3.** Maximal Price for Resmetirom by Yearly Treatment Discontinuation Rate at 3 Different Willingness-to-Pay Thresholds

Discontinuation rate	Maximal price of resmetirom		
	$50 000/QALY threshold	$100 000/QALY threshold	$150 000/QALY threshold
0	$5645	$8132	$10 619
0.10	$7182	$10 297	$13 413
0.21^a^	$10 914	$15 406	$19 897
0.30	$13 309	$18 737	$24 165
0.40	$16 311	$22 847	$29 382
0.50	$19 974	$28 181	$36 388

^a^ Base-case value.

### Scenario and Sensitivity Analysis

**Figure 2.**
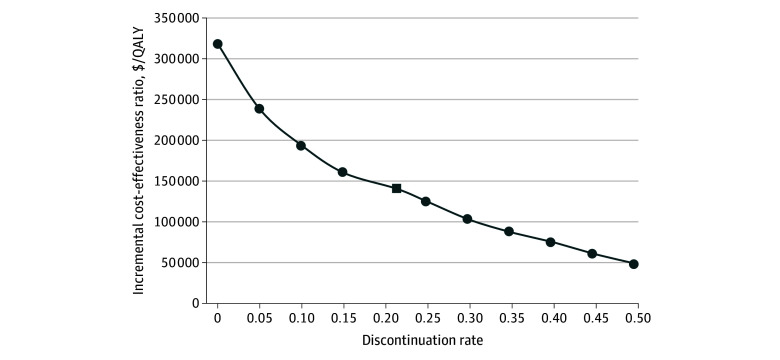
Incremental Cost-Effectiveness Ratio by Annual Discontinuation Rate Box indicates base-case value; QALY, quality-adjusted life-year.

In 1-way sensitivity analysis, the discontinuation rate had a strong impact on ICER. At an annual discontinuation rate of 4.3%, the ICER was $248 970/QALY. Increasing the discontinuation rate lowered drug costs, reduced estimated treatment effectiveness, and increased medical costs, but the ICER also declined (Figure 2). At a discontinuation rate greater than 30.0%, the ICER would be less than $100 000/QALY. Without any discontinuation, the ICER would be $318 740/QALY. To be cost-effective, the annual price could not exceed $5645 to $10 619, depending on the willingness-to-pay threshold (Table 3). Other influential variables included treatment efficacy; utility values for F1, F2, and F3 or MASH-F3; transition probabilities from F3 and F4 (with or without MASH) to HCC; and transitions between MASH-F3 and MASH-F4 (eFigure 2 in Supplement 1). The disutility of taking resmetirom had little effect on the ICER, which ranged from $141 712/QALY to $146 665/QALY. When we substituted transition probabilities of MASLD natural progression based on the placebo groups from RCTs, the ICER was $101 135/QALY. For patients with coexisting MASLD and T2D, the ICER was $86 131/QALY (eTable 3 in Supplement 1).

## Discussion

This study found that compared with SoC, treatment with resmetirom was associated with fewer cases of MASLD-related HCC, LTs, and liver deaths, a savings of $18 499 in MASLD-related medical costs, and medication costs of $54 754/patient over a lifetime, resulting in a discounted incremental cost of $36 255 and an ICER of $140 134/QALY. Depending on the willingness-to-pay threshold—$50 000 to $150 000/QALY—maximal annual drug price varied from $10 914 and $19 897. Of note, to facilitate comparison with previous analyses,^26,27^ our base-case analysis used a drug price of $19 011/year, which was 67% lower than the wholesale price.^10^ It is unrealistic that health insurance plans would pay the wholesale price, which was not cost-effective under any assumptions. They would instead negotiate discounts, as they do for diabetes drugs,^32^ but these prices are proprietary. Because our study aimed to determine the maximal price at which resmetirom could be cost-effective, the base-case drug price did not affect our conclusion.

The cost-effectiveness was sensitive to changes in treatment efficacy, particularly to the efficacy in improving MASH-F3, which changed the ICER by more than 30%. Plausible variations in the efficacy of MASH resolution changed the ICER by 20%. We assumed treatment efficacy remained stable over time and treatment had favorable effects on fibrosis and MASH even after patients moved to fibrosis stages F0 and F1. Long-term clinical trial data will confirm whether this important assumption holds. The ICER was also sensitive to changes in utility of non–MASH-F1 and F2. Because quality-of-life data for people with F1 and F2 without MASH are not available, we assumed their quality of life would be no worse than those with MASH. Further studies on quality of life for these patients are necessary to inform future cost-effectiveness analyses of MASLD treatments. Finally, disease progression could impact cost-effectiveness. At progression rates estimated from RCTs,^16^ resmetirom was more beneficial, with an ICER of $101 135/QALY. However, because patients in RCTs often have more severe disease not representative of the overall population, these progression probabilities are likely overestimated. In patients with coexisting MASLD and T2D, who were assumed to have faster disease progression than those with MASLD alone, the ICER was $86 131/QALY. This suggests that resmetirom may provide greater value in this subgroup, despite the RCT reporting similar efficacy in patients with T2D and the overall trial population—all of whom had well-controlled blood glucose.^8^ Future studies should examine whether disease progression and treatment response in patients with poorly controlled diabetes influence resmetirom’s cost-effectiveness.

Similar to treatments of other chronic diseases, resmetirom might need to be taken for life. One unexpected finding of our study was that the lower the discontinuation rate, the higher the effectiveness and reduction in medical cost, but also the higher the lifetime drug cost. This led to a seemingly counterintuitive finding: the higher the discontinuation rate, the more cost-effective resmetirom became. Because resmetirom rapidly improved fibrosis, while MASLD progressed slowly, lifetime treatment produced only a small incremental health benefit over shorter durations, and this did not justify the high lifetime drug cost. If no patients discontinued treatment, the price would need to be halved from our base-case estimates for resmetirom to remain cost-effective. For example, at a willingness-to-pay threshold of $100 000/QALY, the annual price would need to decrease from $19 011 to $8100. This raises an important question for future study: what is the optimal treatment duration of resmetirom?

In previous studies, the cost-effectiveness of resmetirom ranged from cost-saving to cost-effective at a $100 000/QALY willingness-to-pay threshold.^26,27^ An analysis by the Institute for Clinical and Economic Review (hereafter, the institute) showed that resmetirom would be cost-saving at a price of $19 011/year.^13,27^ Another study conducted by the resmetirom manufacturer reported an ICER of $53 929/QALY and estimated the maximum price of $34 202/year at a willingness-to-pay threshold of $150 000/QALY.^26^ Although both the institute’s and manufacturer’s studies used cohort models with a similar structure regarding MASLD progression and non-MASLD complications (ie, CVD protection), the manufacturer’s study included a slightly younger population (aged 50 vs 55 years) with more than 50% of patients having F1, a population not approved for treatment by the FDA. It also relied on phase 2 data for treatment efficacy, accounted for some treatment-related adverse events, and used much lower medical costs for fibrosis stages F0 to F3, DC, and post-LT, while applying a higher cost for LT than the institute’s model. In addition, they used fixed rather than time-variable transitions from DC and HCC to LT. These major differences led to different ICERs, although both studies found resmetirom cost-effective at the suggested price. In contrast, our study used slower transition probabilities, which were estimated from an updated meta-analysis^17^ of liver biopsies and allowed transitions between MASH and non-MASH health states. This represents a more realistic natural history model structure that was well-validated against longitudinal data. In addition, we applied treatment efficacy of MASH resolution and excluded any cardiovascular benefit due to lack of clinical data. Including cardiovascular benefits would further improve the cost-effectiveness, but long-term follow-up data are needed to determine whether LDL reduction with resmetirom translates into CVD reduction.

### Limitations

Our study has several limitations. First, the natural history of MASLD is complex, and much remains unknown about its progression.^2^ Our model used the latest natural history data and was validated against NASH CRN data. However, conflicting data regarding the risk of MASLD-related HCC may affect its accuracy.^23,38^ We estimated the impact of resmetirom based on natural history, which could be inaccurate. For example, patients who progress and then regress to F2 or MASH-F2 might respond to treatment differently from those who simply remain in F2. Second, in the absence of long-term data, we assumed that treatment efficacy remained constant and that patients with a fibrosis stage of F1 or lower would experience the same treatment benefit as patients with MASH-F2. This represents a best-case scenario. If future studies find no efficacy in a population with less severe disease or if efficacy declines over time, the drug price would need to be reduced further to maintain cost-effectiveness. Another key assumption was that patients with fibrosis without MASH would experience benefits similar to those with MASH. Recent RCTs have only included patients with fibrosis and MASH, so effectiveness in patients without MASH is unknown. However, observational studies suggest that fibrosis, rather than MASH, predicts mortality.^39,40^ Future RCTs should consider enrolling patients without MASH to better understand treatment effectiveness in this population. We also assumed a constant discontinuation rate, which might not be realistic. Patients who persist with therapy probably have not experienced severe adverse effects and may be more affluent than those who discontinue. Additionally, we assumed that simulated patients received nutrition and exercise counseling as provided in the RCT. Variable adherence to lifestyle changes outside of an RCT could impact the cost-effectiveness. However, limited data on the interaction between lifestyle and resmetirom’s effectiveness makes it challenging to explicitly model diet and exercise. Future real-world data will help to evaluate resmetirom’s effectiveness in broader patient populations with varying engagement in lifestyle interventions.

## Conclusions

In this economic evaluation, we found that resmetirom was not cost-effective at a $100 000/QALY threshold, given base-case assumptions regarding its long-term benefits, discontinuation rate, and drug price ($19 011/year), but it could be cost-effective at a lower price. The conclusion was sensitive to changes in discontinuation rate, with higher rates making resmetirom more cost-effective. Further data on the drug’s long-term efficacy and potential benefits on non-MASLD complications would be necessary to accurately determine the economic value of resmetirom.

## Notes

Supplement 1. **eAppendix.** Supplementary Methods **eTable 1.** Association Between Type 2 Diabetes Status and Fibrosis Progression Among Participants in the NASH-CRN Database **eTable 2.** Association Between Type 2 Diabetes Status and NASH Development Among Participants in the NASH-CRN Database **eTable 3.** Scenario Analysis: Discounted Lifetime Costs and Effectiveness of Treatment With Resmetirom in Patients With MASH-F2 or MASH-F3 and Type 2 Diabetes **eFigure 1.** Model Validation: Survival Curve Estimated by Our Model Compared With Observed Survival Among NASH CRN Participants **eFigure 2.** Tornado Diagram for 1-Way Sensitivity Analysis of Model Inputs

Supplement 2.
Data Sharing Statement
