# Supplementary material for: Value-Based Pricing of Resmetirom for Metabolic Dysfunction–Associated Steatotic Liver Disease
Source: JAMA Netw Open. 2025 Jun 27;8(6):e2517122. doi: 10.1001/jamanetworkopen.2025.17122 (PMC12205400; doi:10.1001/jamanetworkopen.2025.17122)
Supplement: Supplement 1. — eAppendix. Supplementary Methods eTable 1. Association Between Type 2 Diabetes Status and Fibrosis Progression Among Participants in the NASH-CRN Database eTable 2. Association Between Type 2 Diabetes Status and NASH Development Among Participants in the NASH-CRN Database eTable 3. Scenario Analysis: Discounted Lifetime Costs and Effectiveness of Treatment With Resmetirom in Patients With MASH-F2 or MASH-F3 and Type 2 Diabetes eFigure 1. Model Validation: Survival Curve Estimated by Our Model Compared With Observed Survival Among NASH CRN Participants eFigure 2. Tornado Diagram for 1-Way Sensitivity Analysis of Model Inputs [file jamanetwopen-e2517122-s001.pdf]

Supplemental Online Content

Le P, Dasarathy S, Herman WH, et al. Value-based pricing of resmetirom for metabolic dysfunction–associated steatotic liver disease. *JAMA Netw Open.* 2025;8(6):e2517122. doi:10.1001/jamanetworkopen.2025.17122

eAppendix. Supplementary Methods

eTable 1. Association Between Type 2 Diabetes Status and Fibrosis Progression Among Participants in the NASH-CRN Database

eTable 2. Association Between Type 2 Diabetes Status and NASH Development Among Participants in the NASH-CRN Database

eTable 3. Scenario Analysis: Discounted Lifetime Costs and Effectiveness of Treatment With Resmetirom in Patients With MASH-F2 or MASH-F3 and Type 2 Diabetes

eFigure 1. Model Validation: Survival Curve Estimated by Our Model Compared With Observed Survival Among NASH CRN Participants

eFigure 2. Tornado Diagram for 1-Way Sensitivity Analysis of Model Inputs

This supplemental material has been provided by the authors to give readers additional information about their work.

© 2025 Le P et al. *JAMA Network Open*.

eAppendix. Supplementary Methods

Scenario analysis

Patients with coexiting MASLD and type 2 diabetes (T2D) often have higher risks of advanced liver complications than those with MASLD and without T2D. We conducted a scenario analyis assuming a faster disease progression for patients with MASLD and T2D than the natural history. Specifically, we used the NASH-CRN database to fit two Cox proportional hazard models, one for fibrosis progression and another one for NASH development. These models were adjusted for demorgraphics (age, sex, race, ethnicity), BMI, baseline fibrosis stage, and T2D status (yes/no). Patients with T2D had an increased risk of fibrosis progression (hazard ratio, HR = 1.62; 95% CI; 1.12-2.34) and NASH development (HR = 2.28; 95% CI; 1.27-4.07) (Supplementary tables 1 & 2). We multiplied the disease transitions in our model by these HRs to reflect faster progression in patients with MASLD and T2D.

© 2025 Le P et al. *JAMA Network Open*.

eTable 1. Association Between Type 2 Diabetes Status and Fibrosis Progression Among Participants in the NASH-CRN Database

	Hazard ratio	95% CI	
Type 2 diabetes (ref. = no)	1.62	1.12	2.34
Age	0.99	0.98	1.01
Male (ref. = female)	0.84	0.58	1.20
White (ref. = non-White)	1.03	0.59	1.79
Hispanic (ref. = not Hispanic)	0.42	0.19	0.94
BMI	0.98	0.96	1.01
Baseline fibrosis stage	0.87	0.74	1.02

© 2025 Le P et al. *JAMA Network Open*.

eTable 2. Association Between Type 2 Diabetes Status and NASH Development Among Participants in the NASH-CRN Database

	Hazard ratio	95% CI	
Type 2 diabetes (ref. = no)	2.28	1.27	4.07
Age	1.00	0.97	1.03
Male (ref. = female)	0.70	0.41	1.19
White (ref. = non-White)	1.72	0.65	4.56
Hispanic (ref. = not Hispanic)	0.96	0.32	2.88
BMI	1.01	0.97	1.06
Baseline fibrosis stage	1.18	0.94	1.49

© 2025 Le P et al. *JAMA Network Open*.

eTable 3. Scenario Analysis: Discounted Lifetime Costs and Effectiveness of Treatment With Resmetirom in Patients With MASH-F2 or MASH-F3 and Type 2 Diabetes

	Medical costs, $	Drug costs, $	Total costs, $	Incremental total costs, $	QALYs	Incremental QALYs	ICER, $/QALY
SoC	140,955	0	140,955		10.03		
Resmetirom	115,995	52,935	168,930	27,975	10.35	0.32	86,131

© 2025 Le P et al. *JAMA Network Open*.

eFigure 1. Model Validation: Survival Curve Estimated by Our Model Compared With Observed Survival Among NASH CRN Participants

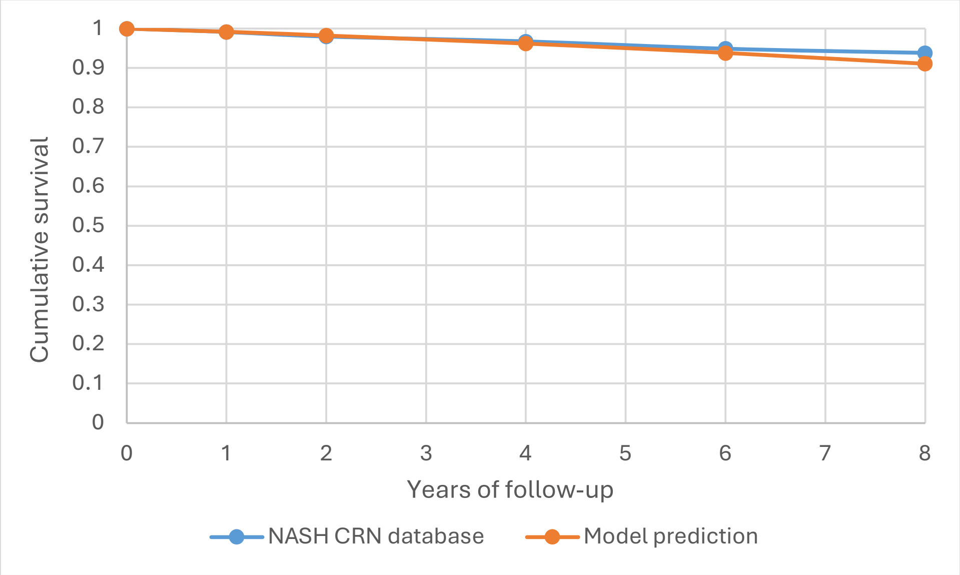

NASH, nonalcoholic steatohepatitis; CRN, clinical research network.

© 2025 Le P et al. *JAMA Network Open*.

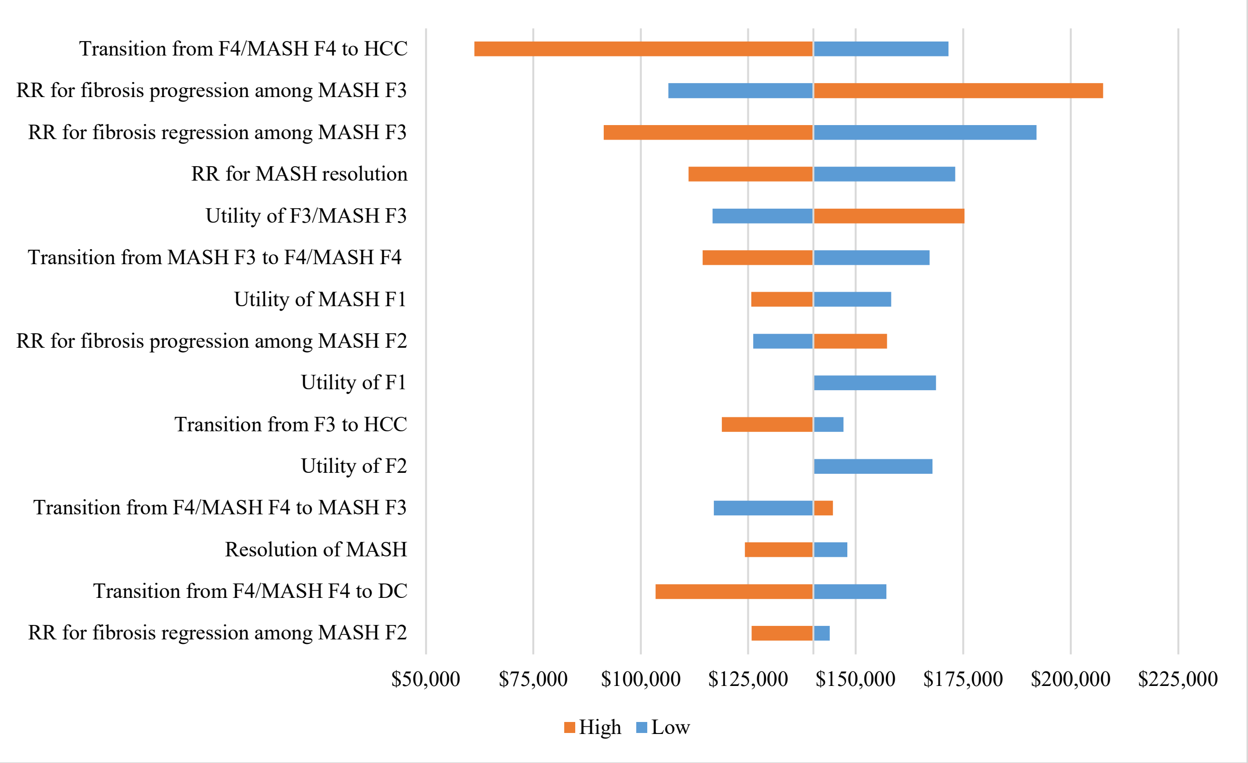

eFigure 2. Tornado Diagram for 1-Way Sensitivity Analysis of Model Inputs

DC, decompensated cirrhosis; F1-F4, fibrosis stage 1-4; HCC, hepatocellular carcinoma; MASH, metabolic dysfunction-associated steatohepatitis; QALY, quality-adjusted life year; RR, relative risk.

© 2025 Le P et al. *JAMA Network Open*.
