# Supplementary material for: Value-Based Pricing of Resmetirom for Metabolic Dysfunction–Associated Steatotic Liver Disease
Source: JAMA Netw Open. 2025 Jun 27;8(6):e2517122. doi: 10.1001/jamanetworkopen.2025.17122 (PMC12205400; doi:10.1001/jamanetworkopen.2025.17122)
Supplement: Supplement 2. — Data Sharing Statement [file jamanetwopen-e2517122-s002.pdf]

Data Sharing Statement

Le. Value-Based Pricing of Resmetirom for Metabolic Dysfunction–Associated Steatotic Liver Disease. *JAMA Netw Open*. Published June 24, 2025.
doi:10.1001/jamanetworkopen.2025.17122

Data
Data available: No

Additional Information
Explanation for why data not available: All data were derived from published literature.
